# Comparing the Impact of Multi-Session Left Dorsolateral Prefrontal and Primary Motor Cortex Neuronavigated Repetitive Transcranial Magnetic Stimulation (nrTMS) on Chronic Pain Patients

**DOI:** 10.3390/brainsci11080961

**Published:** 2021-07-22

**Authors:** Sascha Freigang, Christian Lehner, Shane M. Fresnoza, Kariem Mahdy Ali, Elisabeth Hlavka, Annika Eitler, Istvan Szilagyi, Helmar Bornemann-Cimenti, Hannes Deutschmann, Gernot Reishofer, Anže Berlec, Senta Kurschel-Lackner, Antonio Valentin, Bernhard Sutter, Karla Zaar, Michael Mokry

**Affiliations:** 1 Department of Neurosurgery, Medical University Graz, 8036 Graz, Austria; christian.lehner@medunigraz.at (C.L.); kariem.mahdy-ali@medunigraz.at (K.M.A.); elisabeth.hlavka@stud.medunigraz.at (E.H.); annika.eitler@stud.medunigraz.at (A.E.); Anze.Berlec@uniklinikum.kages.at (A.B.); senta.kurschel@medunigraz.at (S.K.-L.); bernhard.sutter@medunigraz.at (B.S.); Karla.Zaar@klinikum-graz.at (K.Z.); michael.mokry@medunigraz.at (M.M.); 2 Institute of Psychology, University of Graz, 8010 Graz, Austria; shane.fresnoza@uni-graz.at; 3 BioTechMed, 8010 Graz, Austria; 4 Department of Paediatric Surgery, Medical University Graz, 8036 Graz, Austria; istvan.szilagyi@medunigraz.at; 5 Department of Anaesthesiology, Critical Care and Pain Medicine, Medical University Graz, 8036 Graz, Austria; helmar.bornemann@medunigraz.at; 6 Department of Radiology, Clinical Division of Neuroradiology, Vascular and Interventionial Radiology, Medical University of Graz, 8036 Graz, Austria; hannes.deutschmann@medunigraz.at (H.D.); gernot.reishofer@medunigraz.at (G.R.); 7 Department of Basic & Clinical Neuroscience, Institute of Psychiatry, Psychology and Neuroscience, King’s College London, London SE5 9RT, UK; antonio.valentin@kcl.ac.uk

**Keywords:** chronic pain, low back pain, repetitive transcranial magnetic stimulation, neuromodulation, dorsolateral prefrontal cortex, primary motor cortex

## Abstract

Repetitive transcranial stimulation (rTMS) has been shown to produce an analgesic effect and therefore has a potential for treating chronic refractory pain. However, previous studies used various stimulation parameters (including cortical targets), and the best stimulation protocol is not yet identified. The present study investigated the effects of multi-session 20 Hz (2000 pulses) and 5 Hz (1800 pulses) rTMS stimulation of left motor cortex (M1-group) and left dorsolateral prefrontal cortex (DLPFC-group), respectively. The M1-group (*n* = 9) and DLPFC-group (*n* = 7) completed 13 sessions of neuronavigated stimulation, while a Sham-group (*n* = 8) completed seven sessions of placebo stimulation. The outcome was measured using the German Pain Questionnaire (GPQ), Depression, Anxiety and Stress Scale (DASS), and SF-12 questionnaire. Pain perception significantly decreased in the DLPFC-group (38.17%) compared to the M1-group (56.11%) (*p* ≤ 0.001) on the later sessions. Health-related quality of life also improved in the DLPFC-group (40.47) compared to the Sham-group (35.06) (*p* = 0.016), and mental composite summary (*p* = 0.001) in the DLPFC-group (49.12) compared to M1-group (39.46). Stimulation of the left DLPFC resulted in pain relief, while M1 stimulation was not effective. Nonetheless, further studies are needed to identify optimal cortical target sites and stimulation parameters.

## 1. Introduction

Pain is recently redefined by the International Association for the Study of Pain (IASP) as “an unpleasant sensory and emotional experience associated with, or resembling that associated with, actual or potential tissue damage” [1]. Pain is considered chronic if it persists or recurs for more than 3 months regardless of whether it is the sole complaint (chronic primary pain) or secondary to an underlying disease (chronic secondary pain) [2]. Worldwide, chronic pain is one of the leading causes of years lived with disability (YLDs) and reduced quality of life (QoL). In Europe, high prevalence rates were reported for back/neck (40%), hand/arm (22%), and foot/leg (21%) pain [3]. Over the past 30 years, although the prevalence of most diseases showed a pattern of steady decline as measured by age-standardised disability-adjusted life-years (DALYs) rates, chronic low back pain (LBP) remained in the top ten (fourth) causes of DALYs for children and younger adults. Low back pain in childhood predicts low back pain in adult life and is more common in female than male individuals at all ages [4]. As modern medicine extends the population age, it is most likely that the global prevalence of LBP will further increase in the following decades. Therefore, research to develop safe and effective interventions is needed to improve health and alleviate the socioeconomic burden of chronic pain patients.

LBP with unidentifiable pathoanatomical and pathophysiological causes is the most common form of chronic pain condition and is termed non-specific low back pain (NSLBP) [5]. With no specific treatment, management of NSLBP focuses on limiting risk exposure (e.g., lifting heavy objects), patient education, and interventions such as exercise and physical therapy to reduce pain. Current literature also suggests that pharmacological treatments with non-steroidal anti-inflammatory drugs (NSAIDs) and acetaminophen, as well as antidepressants, muscle relaxants, and opioid analgesics are effective for chronic LBP [6,7,8]. Nonetheless, non-pharmacological therapies may fail in some patients, and few trials have investigated their effectiveness [4]. With medications, pain relief is achievable but insufficient because any benefit is likely to be temporary, and symptoms will recur when medication is stopped [9]. Treatment-emergent adverse events (e.g., skeletal muscle relaxants-induced sedation) and long-term use related side effects (e.g., increased risk of vascular events for NSAIDs) are also serious setbacks of pharmacotherapy [10,11]. Moreover, with regard to surgical management, there are still no well-defined clinical practice guidelines related to surgical intervention for chronic LBP in the absence of serious anatomical problems [12].

The most challenging issue in managing chronic pain, including NSLBP, is that the underlying mechanisms are still poorly understood. In NSLBP, pain sensations do not necessarily reflect the presence of a peripheral noxious stimulus because neurons in the pain pathway can be activated by a low threshold, innocuous or non-noxious inputs [13]. The neurobiological cause is thought to be maladaptive plasticity such as central sensitization, which manifests as distort or amplify (hyperalgesia and allodynia), increase degree or duration (after sensations and temporal summation), and spatial extent (expansion of the receptive field), as well as a reduced conditioned pain modulation [13,14]. In chronic pain patients, the prevalent expectation for brain activity is a sustained or enhanced activation of areas already identified for acute pain [15]. For instance, increased functional connectivity between sensorimotor and frontoparietal networks could reflect sustained attention to bodily sensations and hypervigilance to somatic sensations [16,17]. Furthermore, compared with healthy controls, patients also exhibit greater resting-state electroencephalography (EEG) alpha oscillations (8.5–12.5 Hz) at the parietal region, which could be relevant with attenuated sensory information gating and excessive integration of pain-related information [17]. The early evoked magnetic field elicited by stimulation of the painful back is also elevated in very chronic patients [18]. Chronicity-dependent cortical reorganization, regardless of aetiology, is also reported in the primary somatosensory (SI) cortex of chronic pain patients [18,19,20].

In contrast to SI, the evidence of altered structural, organizational, and functional alternation in the primary motor cortex (M1) for neuropathic and non-neuropathic pain conditions is conflicting [21]. However, several studies suggest the association of cortical reorganization of muscle representation in M1 with deficits in postural control, such as impaired anticipatory activation of trunk muscles [22,23]. In addition, similar to SI and other pain-relevant brain regions, enhanced neuronal activity/excitability as measured with transcranial magnetic stimulation (TMS); evoked peripheral muscle potentials (motor evoked potentials or MEPs) are reported in M1 [24]. Enhanced cortical excitability is thought to be secondary to M1 disinhibition as indicated by reduced GABA-mediated short-interval intracortical inhibition (SICI) and cortical silent period (CSP), as well as an increase in the glutamatergic-mediated short-interval intracortical facilitation (SICF) [21,24]. A decrease in the level of thalamic and M1 N-acetylaspartate (NAA) is also considered an index of neuronal depression and altered neuronal-glial interactions in chronic pain patients [25,26]. Higher levels of glutamate/glutamine compounds in the amygdala are also observed in fibromyalgia patients compared to healthy controls [27]. Overall, these studies suggest the potential of the cerebral cortex as a promising target in treating chronic pain.

In the past decades, the development of non-invasive brain stimulation methods such as TMS and several variants of transcranial electrical stimulation allows the identification of the causal role of different cortical regions and neuromodulation of these structures to treat pathological conditions such as chronic pain. Application of repetitive magnetic pulses at a specific frequency (repetitive TMS or rTMS) or in a burst of 3–5 pulses delivered at theta frequencies (theta burst stimulation or TBS) can modulate cortical excitability during and beyond the period of stimulation [28,29]. Induction of neuroplasticity, such as long-term potentiation (LTP) and long-term depression (LTD) at the synaptic level is thought to be the neurophysiological mechanism behind the after-effects of rTMS and TBS paradigms [30]. Several studies applied these paradigms to disrupt or reverse maladaptive and enhanced adaptive neuroplasticity associated with chronic pain. Systematic reviews of rTMS studies for chronic pain with known etiological factors (e.g., fibromyalgia) suggest a beneficial effect of a single or repetitive dose of high-frequency stimulation of M1 [31,32,33]. A meta-analytical study suggests that five-sessions of high-frequency (5, 10, and 20 Hz) rTMS on M1 has a maximal analgesic effect lasting up to 1 month in chronic neuropathic pain patients [34]. However, another meta-analysis reported that low-frequency rTMS is ineffective in treating chronic pain, while single doses of high-frequency rTMS of M1 are considered to have no clinical significance for chronic pain due to its negligible effect [35]. On the other hand, the evidence is still insufficient for chronic pain of unknown origin, such as NSLBP. So far, only one study has shown that one week of 20 Hz rTMS applied to the left M1/S1 hand area can decrease pain perception. Significant reduction in visual analogue scale (VAS) and Short Form McGill pain questionnaire (SF-MPQ) scores were observed in the rTMS-treated group but not in the sham group, as well as lower mean pain score compared to patients treated with physical therapy [36].

This study was undertaken to explore the efficacy of rTMS for NSLBP and improve the available protocol in managing chronic pain. We aimed to replicate the beneficial effect of multi-session left M1 rTMS in the study of Ambriz-Tututi and colleagues (2016). In addition to M1, we also applied neuronavigated rTMS (nrTMS) on the left dorsolateral prefrontal cortex (DLPFC) because stimulation of this brain area is reported to change pain perception in healthy subjects and has analgesic effects in acute postoperative pain, fibromyalgia, and traumatic spinal cord injury patients [37,38,39]. Therefore, we hypothesized that left M1 and DLPFC stimulation would reduce pain perception in NSLBP patients. To our knowledge, this is so far the first report exploring the effect of left DLPFC and M1 nrTMS in NSLBP patients in a single study.

## 2. Materials and Methods

### 2.1. Patients

Thirty-four chronic pain patients participated in the study (19 females and 15 males, mean age ± SD: 54 ± 11 years). They were either previous neurosurgical patients or regular pain clinic patients at the University Hospital Graz-Austria. All have no prior knowledge about TMS and had no planned pain-related interventions during the study. The sample size was a priori calculated using G *Power 3.1.9 and is based on a planned repeated measure ANOVA (with within-between interaction) on the numerical pain scale data. We expected an effect size of *d* = 0.20, power = 0.95, and *a* = 0.05. Inclusion criteria were age between 18 and 80 years, clinical diagnosis of chronic LBP and or neck pain, average resting pain-level greater than 3 in the Numeric Rating Scale (0–10), no changes in pain medication 4 weeks before baseline measurements, and no single or multiple surgical procedures in the head and lower back in the last two years. Patients with the following characteristics were excluded from the study: metallic and electronic implants in the head, neck and chest; intake of opioid analgesics (>100 mg orally per day), tetracyclic antidepressants, antiviral, and antipsychotic drugs; history of frequent headache or tinnitus and alcohol or drug abuse; confirmed or suspected pregnancy and breastfeeding. All participants provided written informed consent before the experimental procedures. The Ethics Committee of the Medical University of Graz approved the study (registration number: 30-459-ex 17/18), and all procedures conform to the Declaration of Helsinki regarding human experimentation.

### 2.2. Study Design and Procedure

**Figure 1:**
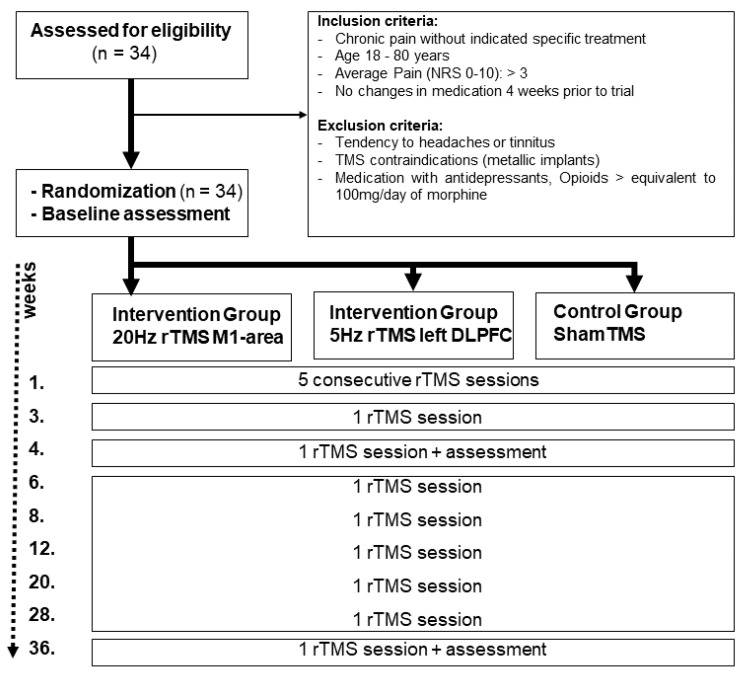
Flowchart depicting the course of the study (CONSORT 2010).

The study was conducted in a single-blinded, randomized, partial placebo-controlled design. It was retrospectively registered at clinicaltrails.gov (registration number: NCT04934150). The experiments took place in the outpatient clinic of the Department of Neurosurgery (Medical University Graz) between February 2019 and March 2020. The patients were allocated into an “M1-group” (5 males, 6 females; mean age ± SD: 53.8 ± 12.7 years), “DLPFC-group” (6 males, 6 females; mean age ± SD: 56.8 + 9.6 years), and “sham-group” (4 males, 7 females; mean age ± SD: 52.5 ± 12.5 years) using permuted block randomization on the online software random.org. Patients were blinded to their assigned group. Each patient in the M1-group and DLPFC-group underwent 13 nrTMS experimental sessions. The first 5 sessions were conducted every day for 5 consecutive days without a break (1 session per day). One week later, the remaining 7 sessions were conducted in a span of 9 months (week 3, 4, 6, 8, 12, 20, 28, and 36). The sham-group followed the same schedule; however, the experiment was stopped after the seventh session (4th week) because of ethical considerations. Each session started with head/brain and TMS coil co-registration. Subsequently, stimulation intensity was determined, and target areas underwent stimulation. Pain assessments before and after stimulation using numerical pain rating scales (NPRS) were conducted on the first (baseline), 7th (4th week), and 13th (36th week) experimental sessions (Figure 1). NPRS scores were documented through an interview before and after stimulation. An experimental session including the preparations lasted for approximately 30 min.

### 2.3. Neuronavigated Repetitive Transcranial Magnetic Stimulation (nrTMS)

**Figure 2:**
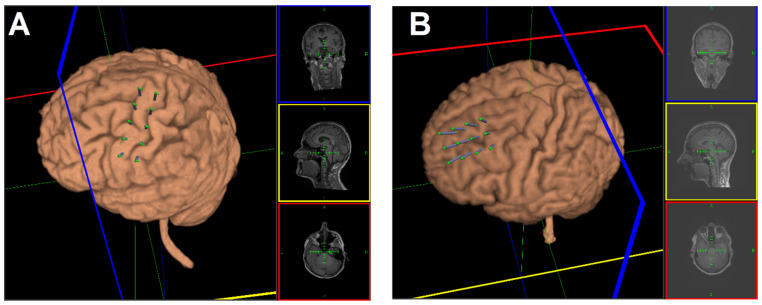
Target arrays for left M1 (**A**) and DLPFC (**B**) rTMS stimulation. The target grid for M1 was a 2 × 5 points array with a 10 mm interpoint distance. For the DLPFC, the target grid was a 3 × 4 points array with a 10 mm interpoint distance. For each participant, the target array was positioned based on anatomical landmarks.

TMS was administered using a figure-of-eight coil (MCF-B65) connected to a MagPro X100 stimulator (MagVenture A/S, Farum, Denmark). Patients were seated on a reclining chair with head and neck support and were asked to relax. For precise coil placement and stimulation, neuronavigation (line-navigated) was performed using Localite TMS Navigator software (LOCALITE Biomedical Visualization Systems GmbH, Sankt Augustin, Germany) that tracts the coil movement with an infrared stereo-optical tracking camera (Polaris Spectra, Northern Digital Inc., Waterloo, Ontario, Canada). The tracking system monitors the location of passive marker spheres attached to the TMS coil and head in real-time. Each patient’s T1-weighted MRI scan (MPRAGE, TR = 1650, TE = 1.82 ms, matrix = 256 × 256, FOV = 256 mm, 192 sagittal slices, in-plane resolution: 1 mm × 1 mm, slice thickness: 1 mm, 0.5 mm gap) was used for head and coil registration, target planning, and neuronavigation during stimulation (Siemens Medical Systems, Erlangen, Germany). TMS parameters were consistent with Ambriz-Tututi et al. (2016): 2000 biphasic pulses at an intensity of 95% resting motor threshold (RMT) applied (10 trains with 28 s inter-train interval (ITI)) for 10 s at 20 Hz. RMT was determined by electromyographic recording over the abductor pollicis brevis muscle and defined as the minimum stimulator output that elicits a 50 uV motor-evoked potential (MEPs) in 5 out of 10 single-pulse TMS stimulation of M1 at rest. Anatomically defined targets over the left M1 were marked by a 5 × 2 grid overlay with 10 mm between-target spacing (Figure 2A), while for the left DLPFC, targets were marked by a 3 × 4 grid overlay (Figure 2B). For the DLPFC-group, 12 trains of 1800 TMS pulses (150 pulses per train, ITI 10 s) were delivered at 5 Hz and 90% RMT [39]. During the stimulations, the coil was held tangentially to the scalp at an angle of 45° to the midsagittal plane generating a current with posterior-anterior direction. TMS was administered over the left M1 in the sham group but with the coil tilted approximately 45 degrees away from the scalp. Therefore, patients could still hear and feel the typical TMS sound and vibration, respectively, without active stimulation. Sham stimulation was limited to seven sessions and used the same frequency, quantity of stimuli, and ITI as the M1-group.

### 2.4. Outcome Assessments

The primary outcome variable was derived from the German Pain Questionnaire (GPQ). At baseline and for each experimental session, patients were asked to verbally rate their perceived greatest pain intensity before stimulation and bearable pain intensity after stimulation using the GPQ-NPRS ranging from 0 (“no pain”) to 10 (“worst pain imaginable”) [40]. As secondary outcome measures, depression, anxiety, and stress scores were obtained using the self-report “Depression, Anxiety, and Stress Scale” (DASS) questionnaire also at baseline, after the 4th and after the 36th week of stimulation [41]. DASS contains 21 items (7 per category: depression, anxiety and stress), and patients were asked to score every item on a scale from 0 to 3 (0 = never, 1 = sometimes, 2 = often, 3 = almost always). The item scores were added, and each category can have a total DASS score of 21. There are separate severity ratings for depression (0 to 4 = normal, 5 to 6 = mild, 7 to 10 = moderate, 11 to 13 = severe, >14 = extremely severe), anxiety (0 to 3 = normal, 4 to 5 = mild, 6 to 7 = moderate, 8 to 9 = severe, >10 = extremely severe), and stress (0 to 7 = normal, 8 to 9 = mild, 10 to 12 = moderate, 13 to 16 = severe, >17 = extremely severe) [42]. To assess the impact of the stimulation on health-related quality of life (HRQoL) measures, the patients answered the 12-item short-form questionnaire (SF-12 version 1) [43]. The SF-12 questionnaire uses eight domains on a 100-point scale which include physical function (PF), role limitations caused by physical problems (RP), pain (BP), general health (GH), vitality/energy (VT), social function (SF), mental health/emotional well-being (MH), and role limitations caused by emotional problems/mental health (RE) [44]. The items refer to perceived health status during the last four weeks; a higher score indicates a better perceived health state. The PF, RP, BP, and GH dimensions were summarized into a physical composite summary (PCS), and the VT, SF, MH, and RE dimensions were summarized into a mental composite summary (MCS) [44]. Unlike the NPRS, DASS and SF-12 scores were only recorded at baseline, after the 4th and 36th week of stimulation.

### 2.5. Statistical Analysis

Statistical analyses were performed using SPSS version 26 software (IBM Corp., Armonk, NY, USA), and figures were generated using Graphpad Prism (GraphPad Prism version 9.0.0 for Windows, GraphPad Software, San Diego, CA, USA). All data were analysed using linear mixed-effects modelling (LMM) with random intercept. We decided to use LMMs for various statistical reasons. First, LMMs are well suited for unbalanced datasets such as ours (sham-group only has a baseline until 4th week measurements, while the M1-group and DLPFC-group have a baseline until 36th week measurements) [45,46]. Second, unlike the regular ANOVA, LMMs does not perform listwise deletion since it has an automatically in-built implicit imputation that assumes the “Missing completely at random (MCAR)” function. For this reason, incomplete data from patients who drop out can still be incorporated into the models. Third, LMMs is a suitable and robust statistical approach because multilevel models tolerate the heterogeneity of variances (due to unequal sizes) between groups. Finally, compared to the traditional ANOVA for a study with repeated measures that only compare the variances between the group means and therefore does not consider the interindividual differences, LMM considers the inter-individual differences by incorporating the participants as a “random factor” in the model. This feature is useful for pain studies because it accounts for the large inter-individual patient’s subjective rating variability.

For the analysis, the GPQ-NPRS, DASS, and SF-12 scores served as the dependent variables. We first analysed GPQ-NPRS data from time points similar to when the DASS and SF-12 scores were obtained (baseline, after 4th and 36th week of stimulation). Before the analysis, the ordinal GPQ-NPRS scores were converted into percentages of the 11-point scale (e.g., “10” = 100% and “0” = 9.09%). The model contained the between-subject factor “group” (sham, M1, and DLPFC) and the within-subject factor “session” (baseline, after 4th and after 36th week of stimulation) and “time” (“before stimulation” = percentage of greatest perceived pain and “after stimulation” = percentage of bearable pain) as fixed factors. This analysis does not give us a complete picture of how multi-session stimulation affected pain intensity; therefore, we plotted baseline and post-stimulation GPQ-NPRS scores from each experimental session and calculated the area under the curve (AUC) using a trapezoidal method in Microsoft Excel (2019). The AUC indicates the summation of pain relief over time. Smaller AUC values indicate a greater decrease in pain intensity. Each patient’s AUC was determined, and the group average was obtained. Using an independent paired *t*-test, we first compared the three groups (sham, M1, and DLPFC) average AUC for the first five days of stimulation. Next, we compared the average AUC from the 4th to the 36th week of stimulation of the M1 and DLPFC-group.

The DASS and SF-12 scores were treated as continuous variables and therefore not converted. For the DASS and SF-12 scores, the model does not contain the within-subject factor “time” since patients only answered these questionnaires after stimulation. Each patient was specified as a random factor (random intercept model) in all models. Normality (data distribution) and homogeneity of variance test were conducted using Shapiro–Wilk and Levene’s test, respectively. In cases of normality violation, all modelled data underwent logarithmic transformation (log10). We also performed a Pearson Chi-Square test to assess the model’s goodness of fit to the data. We calculated Cohen’s *d* as a measure of effect size (<0.2—trivial, >0.2—small, >0.5—medium and >0.8—large). Significant findings from the models were explored with post hoc comparisons (paired *t*-test, two-tailed, Bonferroni adjusted for multiple comparisons). Lastly, we tested collinearity in the final models by determining the tolerance and variance inflation factors. A *p*-value of <0.05 was considered significant for all statistical analyses. All values are expressed as the mean ± standard error of the mean (SEM).

## 3. Results

In total, 34 patients were enrolled in the study (sham- and M1-group: 11, DLPFC-group: 12). Mean age, gender distribution, and chronic pain duration were comparable between the groups (Table 1). Unexpectedly, there were more patients with clinically diagnosed depression in the sham-group than in the treatment groups. All patients tolerated the experimental procedure well. There were no reports of dizziness, headaches, or nausea. However, for reasons unrelated to the stimulation, three patients in the sham-group (all after the first session), two patients in the M1-group (one after the 7th session and one after the 11th session), and five patients in the DLPFC-group (one after the 1st session, one after the 2nd session, one after the 6th session, and two after the 11th session) dropped out from the study. Reasons included demographic challenges such as a longer commute to the treatment facility and the anticipated difficulty following the specific intervals between experimental sessions. Therefore, all modelled data comprised 90.34% of the expected dataset. Shapiro–Wilk test indicates that the NPRS score (*p* ≤ 0.001), DASS scores (depression: *p* ≤ 0.001, anxiety: *p* ≤ 0.001, stress: *p* = 0.032) and SF-12 scores (PCS: *p* = 0.040, MCS: *p* = 0.025) are not normally distributed, hence all were log-transformed. In all models, Levene’s test showed that variances were equal for each group (all *p* > 0.05). Tolerance range and variance inflation factors were equal to 1.000 in the final models indicating that multicollinearity did not affect the findings.

### 3.1. GPQ-NPRS

**Figure 3:**
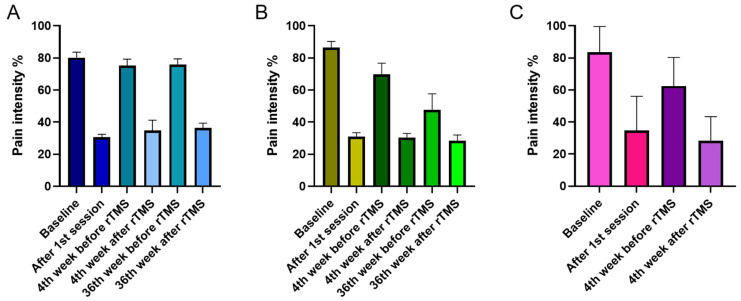
rTMS effects on pain intensity in the M1 (**A**), DLPFC (**B**), and sham group (**C**). The y-axis indicated the mean pain intensity expressed as a percentage (%). The x-axis indicates the time points (in weeks) of conducted measurements. Error bars depict the standard error of mean (SEM).

**Figure 4:**
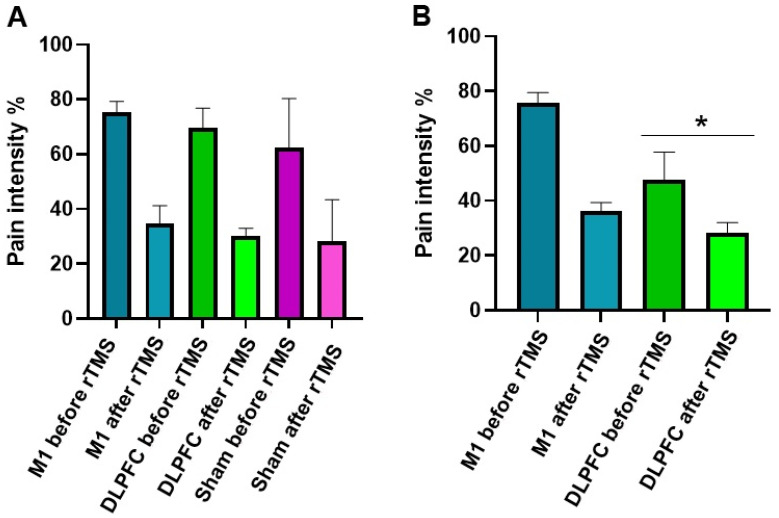
rTMS effects on pain intensity between the groups on the 4th week (**A**) and 36th week (**B**). The y-axis indicates the mean pain intensity expressed as a percentage (%). The x-axis depicts the time points (in weeks) of conducted measurements. The * symbols indicate significant group differences (Bonferroni corrected, two-tailed, paired *t*-test, *p* ≤ 0.05). Error bars depict the standard error of mean (SEM).

For the GPQ-NPRS, we interpreted a full model because the Pearson Chi-Square test indicated the goodness of fit of this model to the data [X^2^ (147) = 4.034, *p* = 0.058]. The results showed that overall pain intensity decreases after rTMS stimulation as indicated by the significant main effect of factor time (*F* (1, 122.52) = 194.14, *p* ≤ 0.001, *d* = 0.961) and interaction of time and session (*F* (2, 122.52) = 3.73, *p* = 0.027, *d* = 0.440) (Figure 3A–C). The overall reduction in pain intensity was significant toward the end of the study as indicated by the factor session’s significant main effect (*F* (2, 134.25) = 4.77, *p* = 0.010, *d* = 0.527). Overall pain intensity on the 4th week (50.19%, *p* = 0.086) of stimulation was comparable to baseline (57.72%), while pain intensity on the 36th week of stimulation was significantly lower (47.19%, *p* = 0.033) than baseline. The main effect of the factor group was only nearly significant (Table 2); however, its interaction with the factor session was significant (*F* (3, 133.89) = 3.99, *p* = 0.009, *d* = 0.457). Bonferroni corrected post hoc *t*-test showed that at baseline and 4th week after stimulation, there were no significant differences in overall pain intensity between the groups (Figure 4A). However, on the 36th week, pain intensity in the DLPFC-group (38.17%) was significantly lower (*p* ≤ 0.001) than in the M1-group (56.11%) (Figure 4B). Additional exploratory post hoc *t*-test showed that the significant differences between the DLPFC- and M1-group’s pain intensity on the 36th week was driven by the significantly lower (*p* ≤ 0.001) pain intensity in the former (47.83%) than the latter (75.81%) before stimulation (Figure 4B). After stimulation, although pain intensity was still lower in the DLPFC-group (28.51%) than the M1-group (36.41%), the differences did not reach significance (*p* = 0.085).

**Figure 5:**
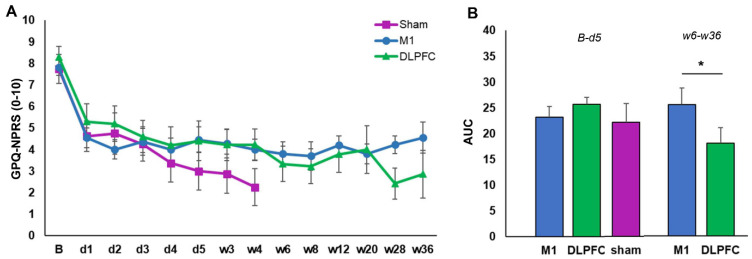
rTMS effects on pain intensity. (**A**) Mean GPQ-NPRS across sessions and stimulation conditions. (**B**) The area under the curve (AUC) across stimulation conditions from baseline up to the 5th day of stimulation (left) and from the 6th week until the 36th of stimulation (right). B  =  baseline; d  =  day; w = week; GPQ-NPRS = German Pain Questionnaire-Numerical Pain Rating scale; M1 = primary motor cortex; DLPFC = dorsolateral prefrontal cortex; Error bars denote standard error of mean (SEM). The * symbol indicates significant group differences (*p* ≤ 0.05).

For the AUC, there were no significant group differences (all *p* ≥ 0.05) from baseline until the end of the daily (5th day) experimental session (Figure 5B). In contrast, the AUC from the 6th to the 13th experimental session was significantly smaller in the DLPFC-group compared to the M1-group (*p* = 0.007) (Figure 5B).

### 3.2. DASS

**Figure 6:**
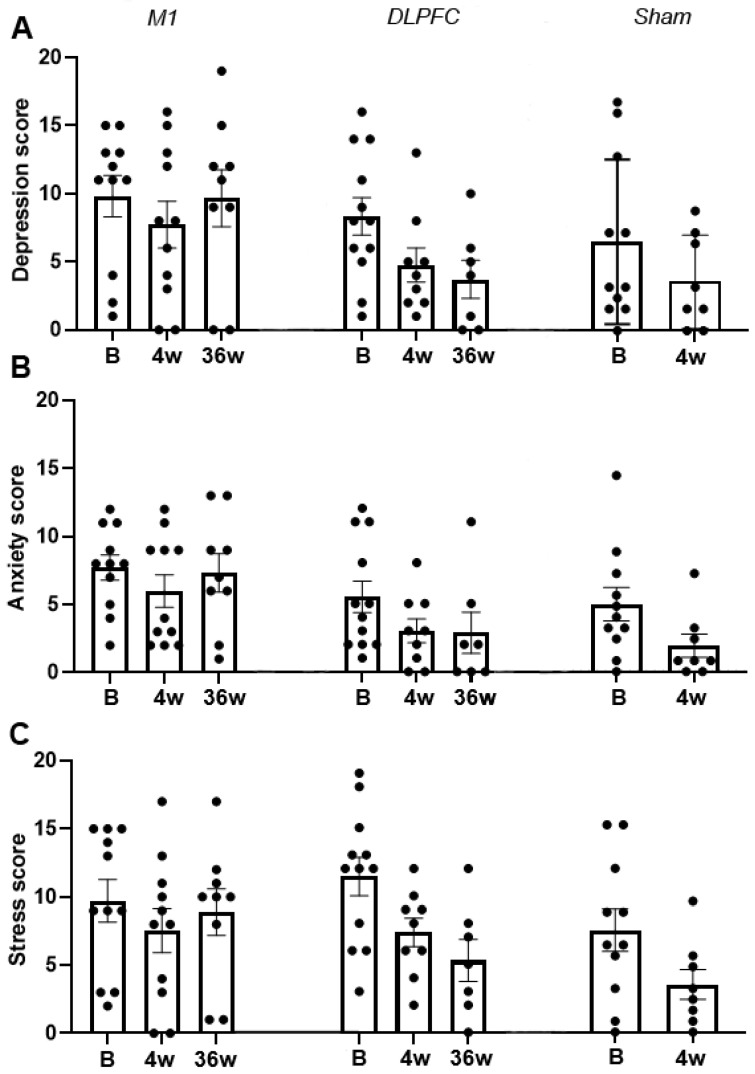
rTMS effects on DASS score for depression (**A**), anxiety (**B**), and stress (**C**). The y-axis indicates the mean score, and the x-axis depicts the time points (in weeks) of conducted measurements. Each dot shows the individual patients’ score. B  =  baseline; w = week; M1 = primary motor cortex; DLPFC = dorsolateral prefrontal cortex; Error bars depict the standard error of mean (SEM).

Pearson Chi-Square test results indicated goodness of fit of the models for the depression score [X^2^ (61) = 7.043, *p* = 0.115], anxiety score [X^2^ (62) = 6.851, *p* = 0.111], and stress score [X^2^ (65) = 6.809, *p* = 0.105]. For the depression score, the model revealed no significant results (Table 2). In contrast, the main effect of the factor session on the anxiety score was significant (*F* (2, 43.19) = 6.90, *p* = 0.003, *d* = 0.433). Bonferroni corrected post hoc *t*-test showed a significant decrease (*p* = 0.001) in anxiety score on the 4th week (4.09) of stimulation compared to baseline (6.47). This indicates a change of overall anxiety level from moderate to mild (Figure 6B). The main effect of the factor session on the stress score was also significant (*F* (2, 40.91) = 12.19, *p* ≤ 0.001, *d* = 0.315). Bonferroni corrected post hoc *t*-test showed a significant decrease in stress score on the 4th week (6.84, *p* = 0.001) and 36th week (7.51, *p* = 0.018) compared to baseline (10.17). These results indicate a change in overall stress level from moderate at baseline to normal on the 4th and 36th week of stimulation (Figure 6C).

### 3.3. SF-12

**Figure 7:**
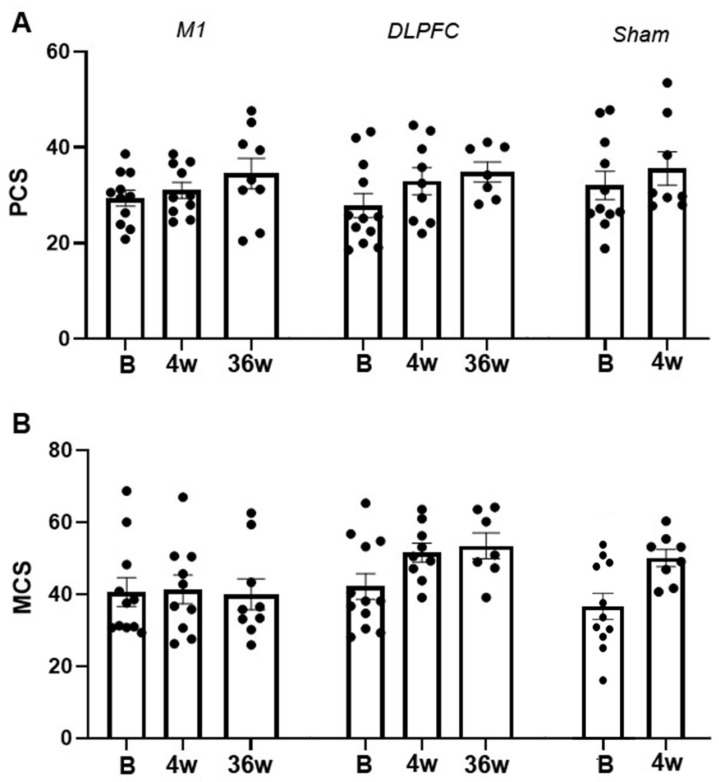
rTMS effects on SF-12 physical composite summary (**A**) and mental composite summary (**B**). The y-axis indicates the mean score, and the x-axis indicates the time points (in weeks) of conducted measurements. Each dot indicates the individual patients’ summary. PCS = physical composite summary; MCS = mental composite summary; B  =  baseline; w = week; M1 = primary motor cortex; DLPFC = dorsolateral prefrontal cortex; Error bars denote mean  ±  SEM.

For the SF-12 questionnaire, we initially modelled PCS and MCS, separately. However, Pearson Chi-Square test indicated that the goodness of fit of this model to the PCS [X^2^ (69) = 0.820, *p* = 0.012] and MCS [X^2^ (69) = 0.955, *p* = 0.014] data were violated. Thus, we modelled the two data sets together. In this full model, the between-subject factor “group” (sham, M1, and DLPFC) and the within-subject factor “session” (baseline, after 4th and after 36th week), and “composite summary” (physical and mental) are the fixed factors. The goodness-of-fit of the full model is better than individual models [X^2^ (140) = 16435.639, *p* ≥ 0.999]. The results of the full model showed a significant main effect of group (*F* (2, 154) = 3.75, *p* = 0.026, *d* = 0.213). Bonferroni corrected post hoc *t*-test indicated a significantly higher (*p* = 0.016) overall score in the DLPFC-group (40.47) than the sham group (35.06). The main effect of session was also significant (*F* (2, 154) = 3.36, *p* = 0.002, *d* = 0.456) as indicated by the significantly higher overall score in the 4th (39.34, *p* = 0.012) and 36th week (40.69, *p* = 0.031) compared to baseline (35.87). Similarly, the main effect of composite summary was significant (*F* (1, 154) = 61.65, *p* ≤ 0.001, *d* = 0.642) as indicated by the significantly higher (*p* ≤ 0.001) MCS (44.89) than PCS (31.86) (Figure 7A,B). Additional exploratory post hoc *t*-test showed that the significantly higher (*p* = 0.001) MCS (Figure 7B) in the DLPFC-group (49.12) than the M1-group (39.46) is the one driving the marginally significant interaction effect of group x composite summary (*F* (2, 154) = 2.46, *p* = 0.089, *d* = 0.241).

## 4. Discussion

This study investigated the potential of multi-session high-frequency rTMS over M1 and DLPFC to treat chronic pain patients. To our knowledge, this is the first investigation of analgesic effects of rTMS applied to these brain regions using an MRI-guided neuronavigation method in a single study. In the active treatment groups (M1 and DLPFC-group), we monitored changes in the sensory-discriminative aspect of pain utilising the GPQ and affective/emotional aspect using the DASS and SF-12 questionnaires for 36 weeks. Assessment in the Sham-group ended after 4 weeks. Compared to left M1 stimulation, the results showed that 36 weeks of left DLPFC stimulation could reduce pain perception and improve health-related quality of life.

In the earlier periods of stimulation, pain perception is reduced as indicated by the reduction in pain intensity scale at baseline and on the 4th week post-stimulation, as well as by the decrease in the AUC covering the first five days of stimulation. However, the reduction in pain perception was comparable between the three groups, which can be due to a robust placebo effect in the sham group or minimal effect of active stimulation in the M1 and DLPFC-group. Robust placebo effect cannot be rule out since a non-significant trend in pain reduction is evident in the sham group in the early period of the study. Placebo effect is common in pain therapies and is thought to arise from expectancy-induced analgesia [47,48]. For instance, the initial decline in GPQ scores in all groups from baseline to day 1 can be attributed to expectancy-induced analgesia because single-dose high-frequency rTMS stimulation only has minimal effects for chronic pain [35]. In brain stimulation studies, the placebo effect may also arise from the modulation of pain perception due to attentional bias caused by the clicking sound of the TMS coil, which may distract or pull the patients’ attention away from the pain. Salient stimuli can disengage the patients from pain signals resulting in altered pain ratings and variations in pain responses [49]. This scenario is possible in our sham group since the tilted coil (active sham) produces a clicking sound even at reduced stimulation intensity.

The possibility of M1 and DLPFC stimulation having minimal effects on pain perception during the early sessions, on the other hand, find support from a meta-analysis of pain studies showing that high-frequency multiple-dose rTMS (e.g., five consecutive days of stimulation) only had minor short-term effects on chronic pain [35]. The authors concluded that the effects do not clearly exceed the predetermined threshold of minimal clinical significance. In contrast, four other review papers reported pain improvement after rTMS treatment, especially in M1 [31,32,33,34]. However, the overall findings of these reviews must be taken with caution because of highly variable rTMS parameters and types of targeted pain across trials. Nevertheless, studies that used stimulation parameters similar to our study for M1 stimulation (20 Hz rTMS at 80–90% RMT applied consecutively for five days) reported pain relief in patients with phantom pain [50], irritable bowel syndrome but limited to those who are hypersensitive [51], diabetic neuropathy [52], central pain after stroke [53], orofacial pain [54], and bladder pain syndrome [55]. For LBP, although there were studies included in the reviews that showed pain relief with 1 Hz and 10 Hz rTMS over M1, the evidence for the efficacy of 20 Hz rTMS for treating chronic LBP is only reported by Ambriz-Tututi and colleagues (2016) and therefore still insufficient. Nonetheless, the non-significant trend in pain reduction we observed in the M1-group (relative to sham) could be reminiscent of the effect shown in their study and requires further exploration.

Concerning the effect of stimulation in later sessions, we observed that pain perception reduction is lower in magnitude in the M1-group than the DLPFC-group. For the M1-group, the weaker effect was persistent since pre- and post-stimulation pain perception at baseline, 4th and 36th week has a waxing and waning pattern (Figure 3A), indicating that pain relief was temporary or only within-session. The absence of an observable reduction in the AUC at later sessions in this group was also suggestive of the transient analgesic effect of M1 stimulation. The impact of repeated M1 stimulation (13 sessions) on pain, particularly in later sessions, is difficult to reconcile with the findings of previous reviews since no study aside from Ambriz-Tututi et al. (2016) have the same experimental design. Nonetheless, the results in the M1-group resemble the short-term reduction in pain intensity reported by studies that used single-dose high-frequency stimulation [31,35]. This could suggest that a cumulative effect of repeated M1 stimulation was not achieved in our study, which is not in accordance with the significant build-up of analgesic effect shown by Ambriz-Tututi et al. (2016). Our methodological approach and stimulation parameters were comparable to their study; therefore, nonconforming results may have been influenced by sample size differences. Participants in their M1-group (*n* = 28) constituted a relatively larger sample size than in our group (*n* = 11). Alternatively, the effectiveness of rTMS for pain relief can be influenced by pain chronicity [56]. Patients with various pain duration histories may respond differently to the stimulation due to the differences in the degree of motor-cortex reorganization or excitability changes (increased excitability and decreased intracortical inhibition). In principle, patients with an extensive reorganization of trunk-muscle representation in M1 may not be amenable to plastic changes induced by rTMS. This scenario is remote since the mean pain duration of patients in our M1-group (5.2 years) is lower than in their group (7.1 years). An alternative theoretical explanation would be that pain-induced functional remodelling of M1 is already finished in patients with longer chronicity giving rTMS a more stable neuronal network to induce plasticity. In contrast, in patients with shorter pain chronicity, M1 is still undergoing functional remodelling, making it an unstable neuronal network to induce plasticity. Moreover, the duration of the lack of somatosensory input, disuse of the limb, and loss of muscle targets may lead to differential changes in M1 excitability between patients [56].

For the DLPFC-group, pain perception reduction was robust compared to that of the M1-group at later sessions. This was indicated by the significantly lower pain intensity scale compared to baseline and compared to those of the M1 group at the same time point. In the DLPFC-group, although post-stimulation pain perception at baseline, 4th and 36th weeks were comparable, there was an evident decline in the pre-stimulation pain perception suggesting that pain no longer reverts to baseline level after each session (Figure 3B). There was also a steady reduction in AUC, further indicating pain relief over time. In summary, these results suggest that multi-session stimulation of the left DLPFC has a cumulative analgesic effect. The DLPFC plays a role in “keeping pain out of mind” by modulation of the cortico-subcortical and cortico-cortical pathways, employing both somatosensory (non-emotional) areas and areas that process emotionally salient stimuli [49,57]. For example, stimulation of the DLPFC may transynaptically modulate the medial prefrontal cortex (mPFC), the brain region best reflecting high magnitude of back pain and the anterior cingulate (ACC), which is dubbed as the main brain region signalling pain, or emotional pain [58]. Moreover, high left DLPFC activity has been shown to reduce the inter-regional correlation of midbrain and medial thalamic activity through a ‘‘top-down’’ mode of inhibition. Therefore, high-frequency rTMS stimulation of the left DLPFC may dampen the effective connectivity of the midbrain-medial thalamic pathway that convey greater affective reactions [57]. High-frequency rTMS of the left DLPFC is also reported to induce dopamine release in several pain-relevant brain areas, including the ipsilateral ACC, medial orbitofrontal cortex, and caudate nucleus [59,60]. Dopamine can have two possible sites of action: peripheral and central. Basal ganglia dopaminergic activity is involved in pain processing and variations in the emotional aspects of pain stimuli, the nigrostriatal dopamine D2 receptor activation to the sensory aspect of pain, while mesolimbic dopamine D2/D3 receptor activity is related to negative affect and fear [49,61,62]. Peripherally, dopaminergic activity may alter pain response due to its potential effect on blood flow and nociception [61,63,64].

Concerning the modulation of pain’s affective/emotional aspect by rTMS, the DASS survey only showed overall improvement in anxiety (moderate to mild) and stress (moderate to normal). A significant change in anxiety level is observed between baseline and the 4th week without group-specificity. For stress, the significant decrease on the 36th week compared to baseline is only driven by the M1 and DLPFC-group since there were no measurements for the sham-group at this period. Although the differences between the groups did not differ statistically, it was evident that the mean stress level on the 36th week was lower in the DLPFC group (6.50) than the M1-group (8.50), indicating normal and mild stress levels, respectively. Improvement in stress level was only present in the DLPFC group, from moderate at baseline to normal on the 36th week. In contrast, in the M1-group, stress levels did not change from baseline to 36th week (both mild). Modulation of brain structures linked to the affective/emotional aspect of pain, such as the cingulate cortex through cortico-subcortical pathways, can directly account for stress level improvement in the DLPFC-group. Imaging studies provided evidence that left DLPFC rTMS also affects blood flow and metabolism in the ACC [65]. The ACC is suggested to be involved in anticipation of pain and higher activity in its anterior and middle segments (including those in the insula) at rest is considered a sign of distorted resting-state network in chronic pain patients [58,66]. Pain anticipation in chronic pain patients is stressful because it is cognitively demanding and may lead to sustained emotional suffering [58]. Reduction in stress level and pain perception in the DLPFC-group may explain why patients in this group (relative to sham) reported a significantly better overall health state in the SF-12 questionnaires. In the 36th week, the DLPFC-group has a superior mental composite summary than the M1-group, which is somehow expected because the emotional and social functioning aspect of pain (vitality/energy, social function, mental health/emotional well-being, and role limitations) is more accessible through DLPFC than M1 stimulation.

## 5. Conclusions

The results of the present study indicate that multi-session rTMS of the left DLPFC leads to significant improvement in pain perception and stress level reduction. These effects are better than those obtained from left M1 stimulation, where no effective pain relief was elicited. This indicates an advantage of the DLPFC as a target area for pain rehabilitation by multi-session rTMS. However, the following limitations of our study must be taken into account. First, there were no measurements from the sham group at later sessions. We considered this a significant drawback of the study because comparisons in those time points are only limited between the M1- and DLPFC-group. In our opinion, data comparisons are not entirely non-trivial because the sham and M1 stimulation (both stimulated left M1) have comparable effects at early time points, while the comparison of data from two separate brain areas (M1 vs. DLPFC) finally revealed significant differences. Second, our sample size was relatively small; hence, further studies with a larger population are warranted. Finally, the patients’ maintenance medications (e.g., selective serotonin and norepinephrine reuptake inhibitor antidepressants (SSNRI) and analgesics) were not discontinued during the study. There are reports that analgesics (e.g., Tramadol) affects cortical excitability [67]. At the same time, serotogenic and adrenergic drugs were shown to modulate plasticity induced by other brain stimulation techniques such as transcranial direct current stimulation (tDCS) and paired associative stimulation (PAS) [68,69]. The impact of these medications on the after-effect of rTMS is unexplored. Still, we cannot entirely rule out their influence on our findings since brain stimulation paradigms share physiological underpinnings. Future studies must replicate the present results in patients who are off-medication at least 24 h before plasticity induction. In conclusion, the present study emphasizes the potential of other pain-related brain regions as treatment targets in chronic pain patients. The study also highlights the importance of brain stimulation methods to investigate the relationship between pain-related brain regions.

## Figures and Tables

**Table 1:** Demographic information of patients who completed the experimental protocols.

	Sham	M1	DLPFC
Number of patients	8	9	7
Mean age (in years)	52.9	51.2	62.6
Gender	F: 2, M: 6	F: 5, M: 4	F: 4, M: 3
Duration of chronic pain (in years)	6.4	5.2	5.3
Patients diagnosed w/depression	6	1	0

**Table 2:** Results of the linear mixed models (LMM) performed for the GPQ-NPRS, DASS, and SF-12 scores.

	Numerator df	Denominator df	*F*-Value	*p*-Value	Cohen’s *d*
**GPQ-NPRS scores**					
Group	2	32.53	3.08	0.059	0.343
Session	2	134.25	4.77	0.010 *	0.527
Time	1	122.52	194.14	<0.001 *	0.961
Group × session	3	133.89	3.99	0.009 *	0.457
Group × time	2	122.52	0.50	0.612	0.251
Session × time	2	122.52	3.73	0.027 *	0.440
Group × session × time	3	122.52	0.55	0.645	0.243
**DASS (depression) score**					
Group	2	30.17	1.43	0.254	0.292
Session	2	35.08	1.76	0.188	0.338
Group × session	3	35.16	1.35	0.274	0.237
**DASS (anxiety) score**					
Group	2	37.03	2.18	0.127	0.295
Session	2	43.19	6.90	0.003 *	0.433
Group × session	3	42.910	0.51	0.678	0.272
**DASS (stress) score**					
Group	2	34.81	0.48	0.625	0.108
Session	2	40.91	12.13	<0.001 *	0.315
Group × session	3	41.04	0.94	0.431	0.141
**SF-12**					
Group	2	154	3.75	0.026 *	0.213
Session	2	154	6.36	0.002 *	0.456
Composite summary	1	154	61.65	<0.001 *	0.642
Group × session	3	154	1.25	0.293	0.222
Group × composite summary	2	154	2.46	0.089	0.241
Session × composite summary	2	154	0.41	0.668	0.359
Group × session × composite summary	3	154	0.221	0.882	0.227

* = indicate significant results (*p* < 0.05), df = Degrees of freedom.

## Data Availability

The data presented in this study are available on request from the corresponding author. The data are not publicly available due to privacy and data protection declaration of the trial.
